# Chronic cough related to the upper airway cough syndrome: one entity but not always the same

**DOI:** 10.1007/s00405-020-06071-y

**Published:** 2020-05-27

**Authors:** Marta Dąbrowska, Magdalena Arcimowicz, Elżbieta M. Grabczak, Olga Truba, Aleksandra Rybka, Katarzyna Białek-Gosk, Karolina Klimowicz, Barbara Jamróz, K. Niemczyk, Rafał Krenke

**Affiliations:** 1 Department of Internal Medicine, Pulmonary Diseases and Allergy, Medical University of Warsaw, Warsaw, Poland; 2 Department of Otorhinolaryngology, Head and Neck Surgery, Medical University of Warsaw, Banacha 1A, 02-097 Warsaw, Poland

**Keywords:** Chronic cough, Upper airway cough syndrome, Allergic rhinitis, Non-allergic rhinitis, Rhinosinusitis

## Abstract

**Purpose:**

Upper airway cough syndrome (UACS), described as chronic cough (CC) associated with allergic (AR), non-allergic rhinitis (NAR) or chronic rhinosinusitis (CRS), is one of the major causes of CC. We aimed to characterize a cohort of UACS patients with special attention to differences between patients with AR and NAR.

**Methods:**

A prospective analysis of clinical data of patients, diagnosed with UACS between 2015 and 2018.

**Results:**

There were 143 patients diagnosed with UACS, median age 52 years, women predominance (68.5%), The group comprised of 59 (41%) AR and 84 (59%) NAR subjects, CRS diagnosed in 17 (12%). Median cough duration: 48 months (IQR 24–120), median cough severity (VAS)—60 mm (IQR 42–78), median Leicester Cough Questionnaire (LCQ) score—11.3 (IQR 8.7–13.7), never-smokers: 70%. The most common symptoms: PND (62%), rhinorrhea (59%), nasal congestion (54%), abnormalities of sinus CT: septum deviation (62%), turbinates hypertrophy (53%), mucosal thickening (53%). UACS as the only cause of CC, was presented in 20 patients (14%). We found no differences between patients with AR and NAR in terms of age, gender, duration and severity of cough, BMI, blood eosinophil count, total IgE and FeNO. AR was associated with higher comorbidity of asthma than NAR (54% vs 35%, *p* = 0.019). Abnormalities in sinus CT scan were more frequently found in patients with NAR than AR (*p* = 0.018).

**Conclusion:**

NAR is the most common upper airway disease associated with UACS. Clinical characteristics of UACS patients with AR and NAR are similar with only minor differences between these groups. It seems reasonable to plan further studies concerning relationship of NAR and cough sensitivity, also in terms of potential similar neurogenic mechanism.

## Introduction

Chronic cough affects 4–10% of the adult population and, in a significant proportion of patients, it may significantly deteriorate quality of life [1, 2]. The most common cause of chronic cough is smoking-related bronchitis, while in non-smoking patients, chronic cough is usually related to gastroesophageal reflux (GER), asthma, upper airway diseases, chronic lung disease and medications, including angiotensin convertase inhibitors (ACEI) [3–6]. Chronic cough associated with different upper airway diseases, such as chronic allergic rhinitis (AR), chronic non-allergic rhinitis (NAR) and chronic rhinosinusitis is referred to as upper airway cough syndrome (UACS) [3–7]. In non-smoking patients, UACS is usually ranked as the first or second leading cause of chronic cough all over the world [5].

Chronic rhinitis or rhinosinusitis has been shown to be an independent risk factor for the development of chronic cough [8]. However, the exact mechanisms of chronic cough in patients with rhinosinusitis are not completely understood. Initially, the pathogenesis of UACS had been viewed to be closely linked to postnasal drip. This view has been challenged by observations that only a small proportion of patients with post nasal drip complain of chronic cough [9], and, on the other hand, some patients with UACS do not suffer from postnasal drip. Therefore, it is currently assumed that the pathomechanism of UACS is more complex and includes postnasal drip, chronic airway inflammation and sensory neural hypersensitivity [8–12]. Despite some progress in the knowledge on the pathogenesis of UACS, the efficacy of treatment of this condition is still limited, with the main therapeutic options being nasal steroids, antihistamines or decongestants [5, 11–14].

Although UACS is a common cause of chronic cough, there have been only few original studies focused on UACS in adult patients [8, 10, 15–17]. Therefore, the aim of our study was to characterize a cohort of patients with UACS diagnosed and treated in our institution with special attention to differences between patients with AR and NAR.

## Material and methods

### General study design

This study was a part of a larger project on chronic cough (CC) which included all patients diagnosed with CC in our institution between 2009 and 2018. The main inclusion criterion was CC (lasting more than 8 weeks) in a non-smoking (at least for 1 year) adult patient. The causes of CC were diagnosed according to the recommendations of the European Respiratory Society, British Thoracic Society and American College of Chest Physicians [3–6] and included detailed medical history, physical examination and additional investigations as described elsewhere [18]. In this study, only patients diagnosed with UACS between January 2015 and December 2018 were analyzed.

### Patients assessment and definitions

UACS was defined as CC in patients with chronic rhinitis or chronic rhinosinusitis. Diagnosis of rhinitis or rhinosinusitis was made by an ENT specialist (MA). All patients with CC underwent ENT examination with nasal endoscopy. When rhinosinusitis or structural abnormalities were suspected sinus CT scanning was performed. Rhinitis was diagnosed if one or more of the following symptoms were present for more than 4 weeks: nasal congestion, rhinorrhea (anterior and/or posterior), sneezing or itchy nose [19]. Allergic rhinitis (AR) required confirmation of atopy by either skin prick tests or serum allergen specific IgE and correlation of nasal symptoms and results of allergic tests [19–22]. Non-allergic rhinitis (NAR) was diagnosed in patients who presented with symptoms of rhinitis lasting for at least an hour daily without clinical signs of nasal infection and without systemic signs of allergic inflammation (no allergen-specific IgE in serum or negative skin prick tests with aeroallergens) [23]. Diagnosis of chronic rhinosinusitis (CRS) was based on clinical criteria (two or more nasal symptoms lasting ≥ 12 weeks) defined by the European Position Paper in Rhinosinusitis and Nasal Polyps (EPOS) document and confirmed by nasal endoscopy and sinus CT scan, evaluated using the Lund and Mackay score [24, 25]. Sinus CT was performed if the patient declared any of the symptoms: nasal obstruction, post nasal drip, smell dysfunction or facial pain. As sinusitis does not exist without rhinitis and may be caused by both AR and NAR, our patients were divided only into two groups depending on their allergy status. We did not distinguish group of patients with rhinosinusitis as they would be sparse and heterogeneous.

Cough severity and its impact on quality of life were measured by Visual Analogue Scale (VAS) and cough related quality of life questionnaire—Leicester Cough Questionnaire (LCQ).

### Statistical analysis

Data are shown as median and ranges or numbers and percentages. Simple descriptive statistics were used to describe patient demographics. Comparisons between groups were made using either *t*-test or Mann–Whitney *U* test for continuous variables and Chi-square test for categorical variables. A *p* value less than 0.05 was considered statistically significant.

## Results

Two hundred and ninety patients with CC were diagnosed and treated in our department between January 2015 and December 2018. UACS was diagnosed in 143 of these patients (49%). The median age of the patients with UACS was 52 years (IQR 37.5–62.5) and women accounted for 68.5% of the study group (*n* = 98). There were 43 ex-smokers (30%), while the remaining 100 patients (70%) were never smokers.

UACS was the sole cause of CC only in 20 patients (14%), whereas in 123 patients with UACS other diseases related to CC were also diagnosed. GERD and asthma were found in 84 (59%) and 61 (43%) patients, respectively. In 21 patients, less common CC causes were also diagnosed (obstructive sleep apnea in 5 patients, non-asthmatic eosinophilic bronchitis in 3, arrhythmia related cough in 3, swallowing disorders in 3, bronchiectasis in 2, chronic bronchitis in 2, treatment with ACEI in 1, *Bordetella pertussis* infection in 1, *M. intracellulare*-related pulmonary disease in 1 patient).

The median duration of cough was 48 months (IQR 24–120). The median severity of cough measured by VAS was 60 mm (IQR 42–78) and median LCQ score was 11.3 points (IQR 8.7–13.7). Dry cough, productive cough and mixed dry/productive cough was declared by 78 (55%), 39 (27%) and 26 (18%) patients, respectively.

**Fig. 1:**
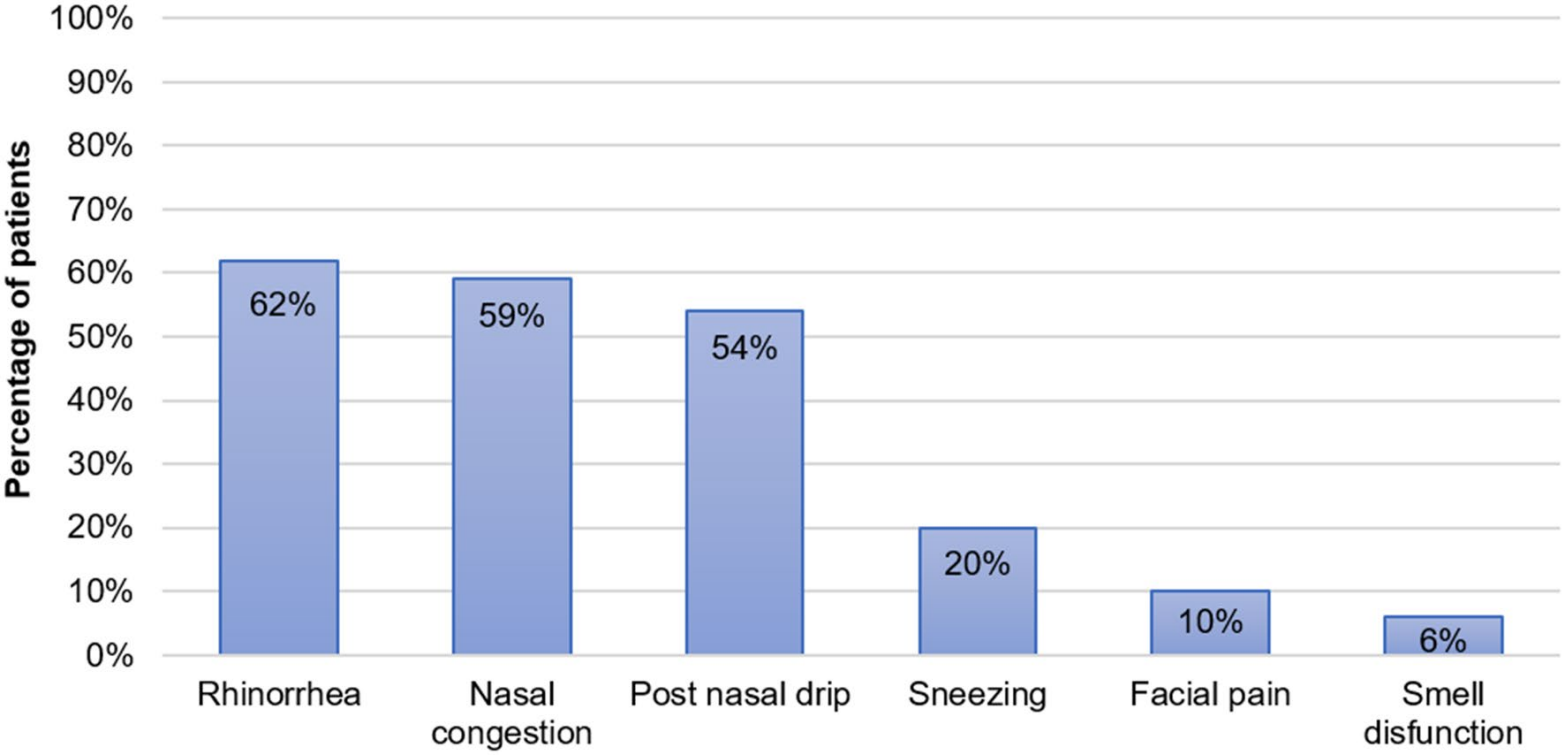
Upper airway symptoms in patients with upper airway cough syndrome

Twenty seven patients reported only one symptom of rhinitis, while the remaining patients reported two or more symptoms. The most common upper airway symptoms were as follows: rhinorrhea (*n* = 88; 62%), nasal congestion (*n* = 84; 59%) and post nasal drip (*n* = 77; 54%). Sneezing or itchy nose, facial pain and smell deterioration were reported by a minority of patients (Fig. 1).

**Fig. 2:**
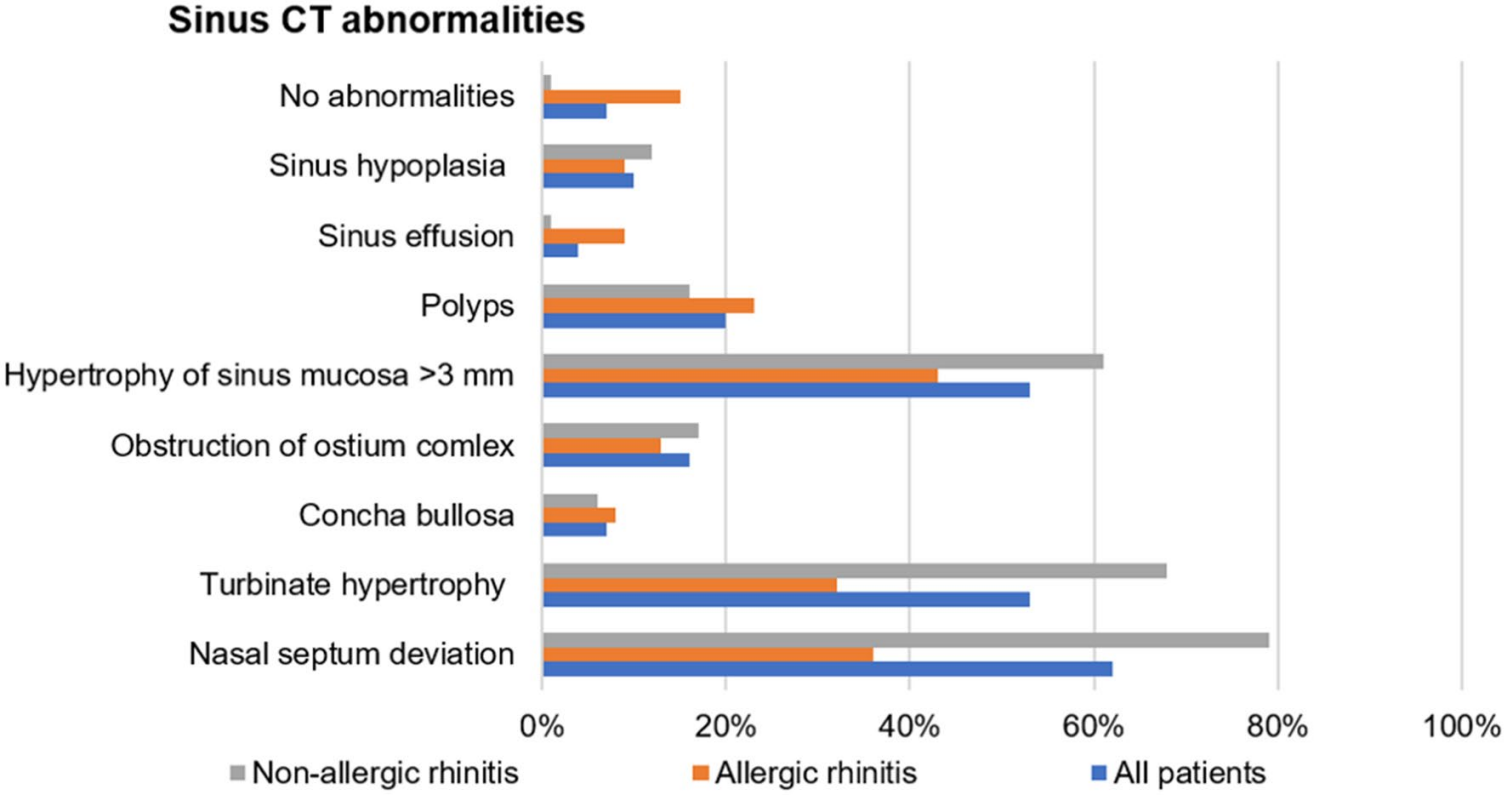
Abnormalities in sinus computed tomography in patients with upper airway cough syndrome

Sinus computed tomography was performed in 116 patients (81%), including 47 and 69 patients with AR and NAR, respectively. Only in eight patients sinus CT showed no abnormalities. The most common abnormalities found in sinus CT scans were: nasal septum deviation (72 patients, 62%), nasal turbinate hypertrophy (62 patients, 53%) and hypertrophy of sinus mucosa (62 patients, 53%). The other abnormalities found in sinus CT are presented in Fig. 2.

**Fig. 3:**
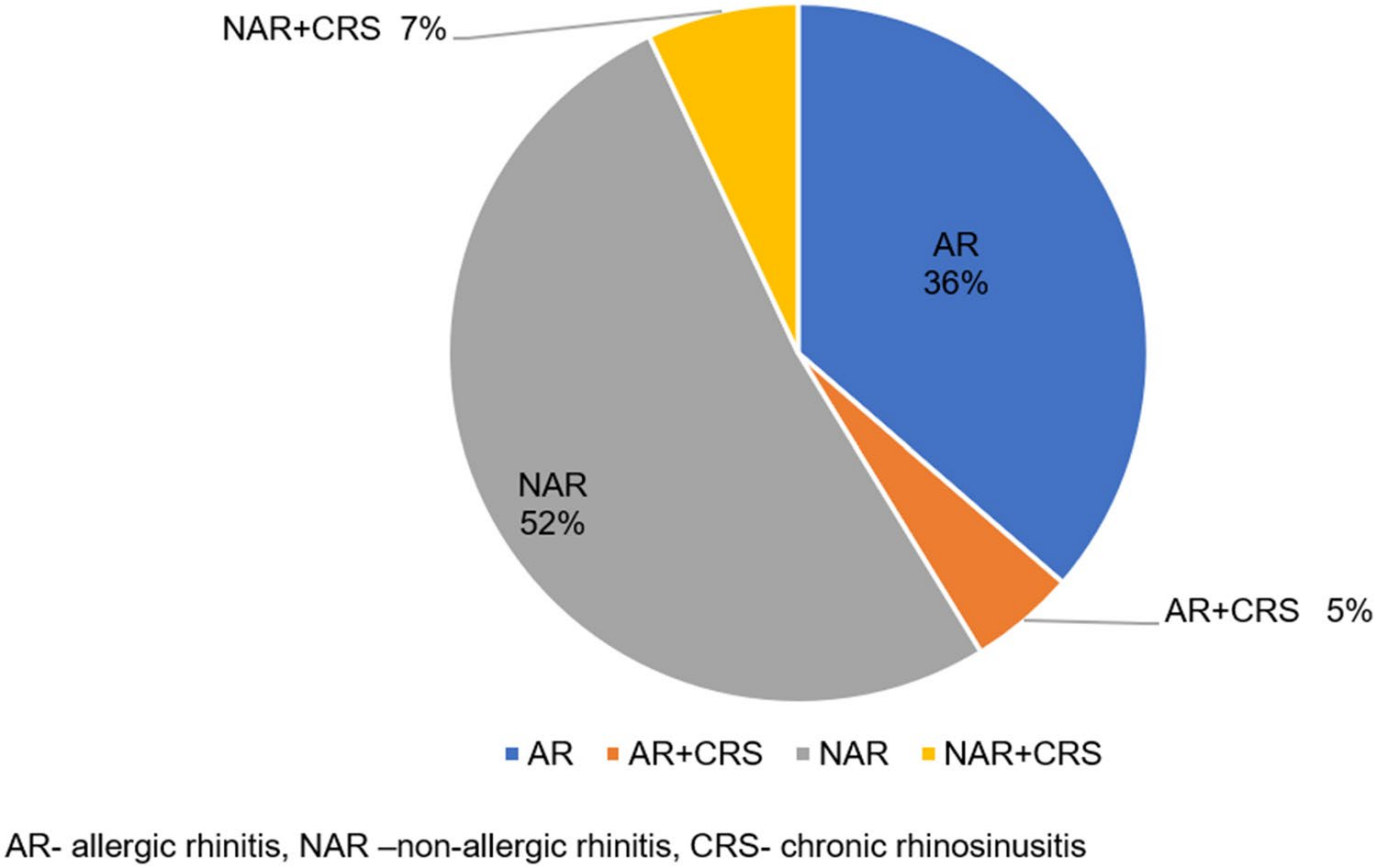
Relative distribution of allergic rhinitis, non-allergic rhinitis and rhinosinusitis in the investigated cohort with chronic cough and upper airway cough syndrome

**Fig. 4:**
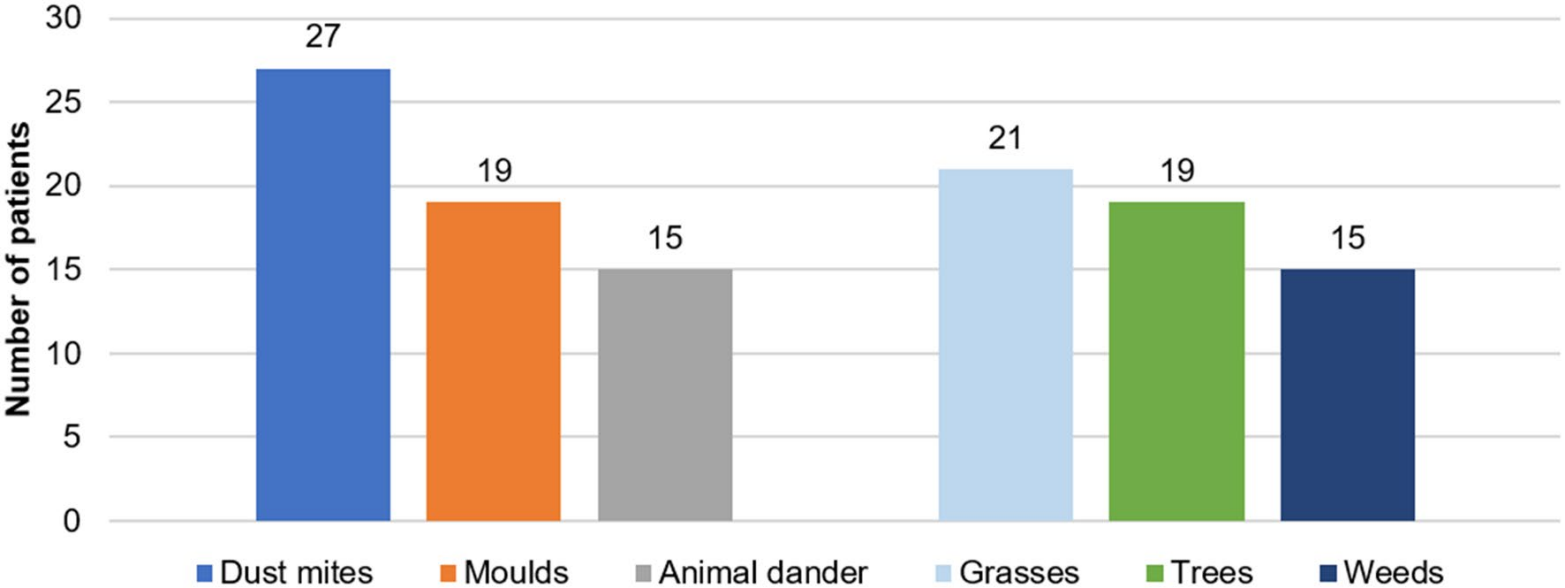
Sensitization to aeroallergens in patients with upper airway cough syndrome due to allergic rhinitis

Allergic rhinitis was diagnosed in 59 (41%), while non-allergic rhinitis in 84 (59%) patients. There were 17 patients (12%) with chronic rhinosinusitis, majority of them due to non-allergic rhinitis (10 patients, 7%) (Fig. 3). Among patients with allergic rhinitis the most common aeroallergens were house dust mite (27/59) and grasses (21/59). Other common allergens are presented in Fig. 4.

Patients with UACS due to AR and NAR did not differ in terms of demographic data, cough characteristics, upper airway symptoms or the results of blood tests (Table 1). Nonetheless, AR was associated with higher comorbidity of asthma than NAR (54% vs 35%, *p* = 0.019). Abnormalities in sinus CT were more frequent in patients with cough due to NAR (Fig. 2).

**Table 1:** Comparison of clinical and biochemical parameters in patients with UACS due to allergic and non-allergic rhinitis

Evaluated data		Allergic rhinitis *N* = 59	Non-allergic rhinits *N* = 84	*p* value
Demographic data	Age (years)	52 (33–61)	56 (38–64)	ns
	Gender F/M	44/15	54/30	ns
	BMI (kg/m^2^)	26.6 (24.4–31.1)	27.3 (23.9–30)	ns
	Smoking history ExS/NS	17/42	26/58	ns
Cough characteristics	Duration of cough (months)	50 (18–128)	48 (24–120)	ns
	Dry/productive/both	35/11/13	43/28/13	ns
	Severity on VAS (mm)	61 (44–73)	59 (42–77)	ns
	LCQ score (points)	11.5 (8.6–13)	11.25 (9.1–13.8)	ns
Symptoms of UACS	Rhinorrhea	38 (64%)	50 (59%)	ns
	Sneezing	11 (19%)	17 (20%)	ns
	Nasal congestion	29 (49%)	55 (65%)	ns
	Facial pain	5 (8%)	8 (10%)	ns
	Smell dysfunction	5 (8%)	3 (4%)	ns
	Post nasal drip	37 (63%)	40 (48%)	ns
Sinus CT abnormalities	Nasal septum deviation	17/47 (36%)	55/69 (79%)	0.00002
	Hypertrophy of sinus mucosa > 3 mm	20/47 (43%)	42/69 (61%)	ns
	Turbinate hyperthrophy	15/47 (32%)	47/69 (68%)	0.0003
	Polyps	11/47 (23%)	11/69 (16%)	ns
	Lund-Mackay score (points)	1 (0–3)	4 (0–6)	ns
Diagnosis of chronic rhinosinusitis		7 (5%)	10 (7%)	ns
Comorbidities	Asthma	32 (54%)	29 (35%)	0.019
	GER	37 (63%)	47 (56%)	ns
	Other	6 (10%)	15 (18%)	ns
	Single cough cause	5 (8%)	15 (18%)	ns
Blood tests	WBC (× 10^3^/uL)	6.0 (5.0–6.9)	6.3 (5.4- 7.1)	ns
	Eos count (per uL)	141 (85–236)	160 (96–256)	ns
	Total IgE (IU/mL)	23.5 (5.3–45.8)	21.5 (10–68)	ns
FeNO	(ppb)	14.9 (11.8–22.7)	13.1 (10.1–24.6)	ns

Data are presented as numbers (and percentages) or median and interquartile range unless otherwise stated

*F* female, *M* male, *ExS* ex-smoker, *NS* non-smoker, *BMI* body mass index, *VAS* visual analogue scale, *LCQ* Leicester Cough Questionnaire, *NAEB* non-asthmatic eosinophilic bronchitis, *WBC* white blood cells, *Eos* eosinophils, *FeNO* fractional exhaled nitric oxide

## Discussion

The results of this study shed more light on various aspects of UACS, including its relative prevalence in patients with CC and anatomical alterations in the nose and paranasal sinuses. To our knowledge, this is one of the very few studies focused on clinical characteristics, e.g., cough severity, in terms of underlying upper airway disease. In this context, the value of this study lies in the relatively large group of patients when comparing to other original studies which included between 21 and 143 subjects [10, 15, 16]. We found that UACS was the most common cause of CC, diagnosed in almost one half of the investigated patients. However, UACS was rarely the sole disease underlying CC (15%), and as many as 85% patients with UACS were diagnosed with other conditions causing CC. We demonstrated that in our investigated group, NAR was the most common upper airway disease associated with UACS. This finding should be emphasized, as the majority of previous studies (both in vitro and in vivo) and reviews on the pathogenesis of UACS were focused on AR or rhinosinusitis [12, 13, 26, 27].

We believe, that in the context of limited effectiveness of CC treatment, new studies which include more specific and in-depth analysis of the underlying disease characteristics are warranted. These studies may help to identify some structural and functional abnormalities or other disease features that may become the targets for more specific therapeutic interventions. This approach is somewhat similar to approach based on the concept of ‘treatable traits’ in patients with asthma and chronic obstructive pulmonary disease. Thus, the importance of the current study is that it is one of the first attempts to more precisely characterize UACS in the context of the specific underlying airway disease and its corresponding CC features.

Anatomical abnormalities of the nose or the paranasal sinuses (nasal septum deviation, hypertrophy of the nasal turbinates, anatomical variations) may contribute to the development of rhinitis/rhinosinusitis, and thus induce UACS [7, 19, 23]. Although structural abnormalities found in sinus CT were common among our patients, their prevalence was not higher than in the general adult population. According to earlier studies, nasal septum deviation and nasal turbinate hypertrophy are both common findings in sinus CT, being present in 48–65% and 42–44% of adults referred to a CT scan due to sinonasal symptoms, respectively [28–30]. Moreover, the relationship between anatomical alterations and upper airway symptoms is equivocal. Ahn et al. showed that albeit nasal septum deviation was demonstrated in 48% of patients, only 3.8% of these patients reported symptoms of nasal congestion [28]. The population in our study was similar to patients in the study by Watelet et al., which focused on patients with upper airway symptoms who also presented with CC [10]. In both studies, nasal obstruction, rhinorrhea and post nasal drip were the predominant symptoms. However, in the study by Watelet et al., 50% of patients had AR, while in our study the majority of patients had NAR and only 12% of patients met the criteria for chronic rhinosinusitis [10].

We did not find significant differences in clinical characteristics between individuals with CC related to allergic or non-allergic rhinitis, except for a higher coexistence of asthma in patients with AR and a higher prevalence of rhinosinusitis due to structural abnormalities in patients with NAR. These associations seem to be easy to understand. Coexistence of AR and asthma has been well documented [20, 31, 32]. Bousquet et al. described the phenomenon of multimorbidity of allergic diseases, such as allergic rhinitis and bronchial asthma and emphasized similar underlying immune and non-immune pathomechanisms in these conditions [33].

The prevalence of UACS in our whole group of patients with CC was quite high (48.6%). Such a tendency has already been observed in our previous studies where UACS contributed to CC in 46% of patients [18]. In other recently published studies the prevalence of UACS is highly variable and ranges from 9 to 82% [5, 34, 35]. The relatively high prevalence of UACS in our study may be at least partially related to the definition of UACS adopted in our study—UACS was diagnosed if chronic cough and at least one symptom of rhinitis were present. Our definition of rhinitis was consistent with the respective definition proposed by experts from ARIA and the American Academy of Allergy, Asthma and Immunology [20–23]. However, it has been clearly demonstrated that the differences in the diagnostic criteria for rhinitis affect its prevalence [36]. It should be emphasized that although in our study only one nasal symptom lasting more than 4 weeks was required to diagnose rhinitis, the majority of our patients reported two or more symptoms. Moreover, signs of chronic inflammation in the nose or the sinuses were confirmed by a dedicated ENT specialist.

It is worth mentioning that in our investigated group, chronic cough was of long duration, intense and bothersome. This may be easily attributed to the profile of our department which is a cough reference center mainly taking care of patients with difficult-to treat cough, who did not respond to previous therapies. This fact could have also resulted in a high percentage of patients with two or multiple cough causes. Therefore, our results may not necessarily be representative for all UACS patients, but only for those with long lasting or difficult-to-treat CC. We suppose that the significant impairment of quality of life in our study might have resulted not only from CC, but from the concomitant upper airway diseases as well. A negative impact of both CC and upper airway diseases on quality of life has been reported in several papers [37–39].

Our study has a few limitations. First, it was a single-center study. Secondly, this study was based on patients referred to “cough clinic”, what may influence on higher severity of CC and upper airway diseases. Third, some data are incomplete, e.g., only 86 patients had LCQ measured, as validation of Polish version of LCQ was available only from 2016.

Despite the above limitations, our study provides detailed data characterizing patients with UACS, and the spectrum of the upper airway disorders leading to the chronic cough. We believe, this may positively impact future therapeutic strategy.

## Conclusion

Upper airway diseases are common cause of chronic cough and they usually coexist with other chronic cough reasons. Among them, the most common is chronic non-allergic rhinitis. The clinical characteristics of UACS patients with allergic and non-allergic rhinitis are similar, with only slight differences between these two groups. Taking our results into consideration, particularly high proportion of patients with NAR, it seems reasonable to plan further studies concerning the relationship of NAR and cough sensitivity, also in terms of a potential similar neurogenic mechanism.
